# IFCC Guidelines (1990) for selection of safe laboratory centrifuges and for their safe use with general purpose appendices concerning centrifuge nomenclature, quantities and units, and calculation of centrifugal acceleration

**DOI:** 10.1155/S1463924691000378

**Published:** 1991

**Authors:** A. Uldall, P. Trier Damgaard, O. Drachmann, F. Jørgensen, D. Kennedy, M. Lauritzen, E. Magnussen, J. C. Rigg, P. Voss

**Affiliations:** 1 University of Copenhagen Department of Clinical Chemistry Herlev Hospital Herlev Ringvej 75, Herlev DK-2730 Denmark


Journal of Automatic Chemistry, Vol. 13, No. 5 (September–October 1991), pp. 221–229

*International Federation of Clinical Chemistry, Scientific Division, Committee on Analytical Systems**

### IFCC Guidelines (1990) for selection of safe laboratory centrifuges and for their safe use

### with general purpose appendices concerning centrifuge nomenclature, quantities and units, and calculation of centrifugal acceleration

**A. Uldall (DK), P. Trier Damgaard (DK), O. Drachmann (DK), F. Jørgensen (DK), D. Kennedy (UK), M. Lauritzen (DK), E. Magnussen (DK), J. C. Rigg (NL) and P. Voss (DK)**

### 1. Introduction

1.1 Safety when operating centrifuges is influenced by three key elements: (1) Selection of centrifuges; (2) Proper operating procedures; and (3) Regular preventative maintenance. The present guidelines focus on these element; the appendices, however, provide guidelines which will be useful in other aspects of centrifugation.

1.2 The mechanical safety of centrifuges available in developed countries has improved within the last 10 years and dangerous situations are nowadays fairly rare. However, accidents still occur — usually in connection with new design of rotor assemblies. Old centrifuges are still being used, both in developed countries, and, especially, in developing countries.

1.3 Several factors need to be considered in the selection of centrifuges: *performance* (for example capacity, start-up time, ease of use, etc.); *safety* (mechanical, electrical, explosion risk if using flammable agents, noise and biological hazards); and *economics*. The International Electrotechnical Commission (IEC) [1, 2] has recently drafted a standard for the safety of laboratory centrifuges; and it is recommended that only those centrifuges meeting the requirements of this standard should be purchased.† Where possible, it is best to select a centrifuge which has been certified as complying with the IEC standard by an independent test house.‡ In the design of centrifuges the risk of expelled parts is usually evaluated, but the IEC document also takes into account the risk of people being harmed by the movement of the centrifuge following a disruption. Accordingly the whole standard requires that a so-called 'clearance envelope' of 300 mm around the centrifuge should be the only space where there is a risk of mechanical hazards outside the centrifuge, including risks from expelled parts and centrifuge movements [1].

1.4 Additional safety requirements to those found in the IEC draft standard are an out-of-balance switch (paragraph 2.8); also, it should be impossible to open the access cover while the rotor is moving. The noise requirements in the IEC standard provide no useful guidelines for selection of centrifuges. Additionally, little is said about centrifuges intended for work with flammable liquids. The present document gives some useful information about these problems.

1.5 These guidelines should be used when no suitable centrifuge, that meets the IEC standard, is available. As a rule of thumb, preference should be given to manufacturers who can supply customers with full preventive maintenance descriptions, monitor mechanical break downs, warn customers of problems and improve designs in the light of users' experience. Generally, by any manufacturer who utilizes a quality management system can do these things [3–7].

1.6 IEC documents relevant to centrifuges give little detail on using a centrifuge safely and the present guidelines may help with this.

*Comments should be sent to: Adam Uldall, Dr Pharm., Ph.D., University of Copenhagen, Department of Clinical Chemistry, Herlev Hospital, Herlev Ringvej 75, DK-2730, Herlev, Denmark.*

* Members: A. Truchaud, Chairman (US); C. A. Burtis, (US); K. Ozawa (JP); H. L. Pardue (US); J. Place (DK); and P. Schnipelsky (US).

† It should be stressed that the IEC standard has yet to be formally accepted and no centrifuge which is recognized as meeting the standard is currently on the market.

‡ This is likely to be based on testing of a single centrifuge, and no running check of the production is mentioned in the draft standard [1]. Therefore it is important to know whether the manufacturer has adopted a quality system as recommended by International Standards Organization (ISO) [3–7], so it is possible to ensure that the centrifuges which are for sale are identical to the one which was investigated by the test house.

© IFCC 1991

1.7 The centrifuges may be classified as in table 1. The present guidelines concentrate on the safety aspects of the types of centrifuges most commonly used in clinical laboratories: small bench-top, bench-top and large-capacity floor-standing centrifuges and high-speed centrifuges. Ultracentrifuges are only briefly covered, but further guidelines can be found elsewhere [8].

1.8 The present publication is the second of two centrifuge guidelines from IFCC. The other is: *Guidelines (1988) for Listing Specifications of Centrifuges* [9]. Both guidelines are based on British Standard 4402: *Specification for safety requirements for laboratory centrifuges 1982* [10], and on a document published in Danish in 1979.

1.9 The proposed nomenclature of centrifuges was first presented in Appendix A to *Guidelines (1988) for Listing Specifications of Centrifuges* [9]. However, against the background of the new standard from the IEC [1, 2], the previously published IFCC-nomenclature has been slightly modified and is presented here as Appendix A. Quantities and units, with regard to centrifuges, are covered in Appendix B. Calculation of the centrifugal acceleration and the kinetic energy of a rotor assembly is described in Appendix C.

### 2. Selection of centrifuges and rotor assemblies on basis of mechanical safety criteria

2.1 The major mechanical hazard of centrifuges originates from the kinetic energy ($E_k$) of the rotor assembly. The manufacturer generally provides figures for the kinetic energy for the maximal rotational frequency; however, the kinetic energy of all accessories and the materials to be centrifuged with the rotor assembly is seldom included. Determination of the kinetic energy of a rotor assembly by experimentation is time-consuming. The kinetic energy is usually estimated through calculation, and complicated formulae based on the geometric construction of the rotor assembly have to be used. However, for rough calculations, the formulae in paragraph 2.1–2.3 Appendix C may be used. The formulae apply to a rotating disk with dimensions that most simulate the actual rotor assembly.

2.2 The choice of centrifuges should be made according to the capacity needed and centrifugal force required, bearing in mind that risks increase with centrifugal force and capacity. In general, centrifuges with low kinetic energy for the rotor assembly are the safest.

2.3 Documentation to support claims made for the strength of the guard barrier, the access cover and the locking system with reference to the kinetic energy of rotor assemblies should be available from the manufacturer. The best documentation consists of a combination of strength calculations and destructive testing investigations. Both types are described in British Standard 4402 [10]. Destructive experiments have shown that even minor changes to the centrifuge design may have a substantial influence on safety [11].

2.4 The access cover lock must be protected against any impact from mechanical failure of the rotor assembly. See also 2.9.

2.5 A sealing gasket between the access cover and the centrifuge chamber must be securely connected, for example by adhesive. The sealing gasket can either be in segments, or be produced of a brittle substance so that a loose strip cannot encircle a test-tube carrier or bucket and tear it out of the trunnions.

2.6 Special facilities or features should be available which eliminate a sudden start up that could cause the buckets to strike the access cover and so be dislodged.

2.7 A centrifuge should not be able to operate at a greater rotational frequency than the mounted rotor can tolerate. A rotational frequency limiting device, or other approaches, should be provided to meet this requirement.

2.8 For a centrifuge with rotor assemblies involving high kinetic energy, for example bench-top centrifuges with $E_k > 1$ kJ and floor-standing centrifuges, an out-of-balance switch is required by law in many countries. This switch cuts the power when a certain degree of imbalance in the rotational parts occurs.

2.9 It should not be possible to open the access cover while the rotor is moving (other than for preventive

*Table 1. Laboratory centrifuges.*

| Type | Rotor assembly | Maximum rotational frequency* min$^{-1}$ (rpm) | Maximum rotational frequency* Hz | Maximum kinetic energy* kJ |
|---|---|---|---|---|
| Small bench-top | Swing-out rotor with 4 × 15 ml samples | 2400 | 40 | 0·1 |
| Bench-top | Swing-out rotor with 8 × 15 ml samples | 3200 | 53·3 | 1·5 |
| Large capacity (floor-standing) | Angle rotor for 6 × 1000 ml samples | 5200 | 86·7 | 73 |
| High speed | Angle rotor for 6 × 300 ml samples | 14 000 | 233 | 150 |
| Ultracentrifuges | Angle rotor for 8 × 35 ml samples | 60 000 | 1000 | 1000 |

* Typical values.

maintenance and repair). For smaller centrifuges (small bench-top centrifuges with i.e. $E_k < 1 kJ$) only a mechanism for cutting the power when opening the lid is required by law in many countries.

2.10 Properly designed centrifuges will keep all debris inside in most mechanical disruptions, however they are seldom foolproof. Therefore, under certain rare circumstances disruptions with expelled parts may occur. Consequently, safety could be increased if each rotor assembly is evaluated separately by experimental disruptions. However, the interaction between the rotor assembly and the rest of the centrifuge during a disruption should always be considered for each rotor likely to be used. In general, the following rules of thumb could be used at an early stage of selection of rotor assemblies:

(*a*) Angle rotors are preferable to swing-out rotors because accidents are most frequently associated with the suspension system of the buckets (but when using an angle rotor tubes should always be stoppered to minimize aerosol production).

(*b*) Light rotor assemblies are preferable to heavy ones. However, the rotor assembly should be able to tolerate about five times the normal stress without breakage.

(*c*) When making a choice from models which provide the same centrifugal force, the centrifuge selected should be that which has the smallest radius — of the rotor assembly — and which revolves at the highest rotational frequency.

(*d*) Generally it is preferable to select swing-out rotor models which have buckets which are suspended in trunnion carriers (for example, a trunnion ring into which the bucket is placed), rather than buckets which have projections that are suspended in the rotor. The construction of the suspension system must, however, be assessed as a whole.

(*e*) Swing-out rotors which are mounted in a rotating inner chamber with an access cover are preferable because if a bucket becomes disengaged it cannot be compressed between the rotor and the outer access cover, or outer casing, thus providing greater mechanical safety. See also 3.1–3.3.

(*f*) Ideally a swing-out rotor should be designed so that centrifugation is impossible when there is a faulty suspension of buckets.

(*g*) Some swing-out rotors have slots into which the corresponding bucket suspension trunnions are designed to engage. To reduce the risk of dislodgement the angle of the slots of the rotor in comparison to a vertical line should slope considerably towards the centre of rotation (figure 1).

(*h*) Buckets of the same type should, as far as possible, contain the same mass and the same mass distribution, in order that numbering is unnecessary.

(*i*) To avoid breakage, and mishaps caused by human error, the range of adaptors for glass holders and racks should be kept as small as possible — preferably all types of glass holders should fit into the bucket, and buckets for each type of racks should be available.

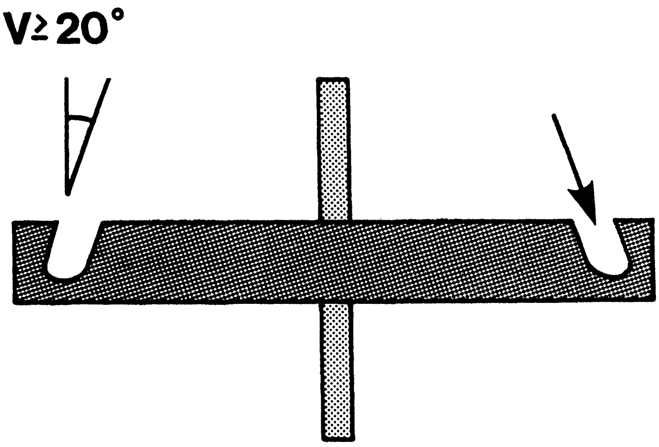

*Figure 1. A rotor with suspension slots. One of these is indicated by an arrow. The slope of the slot in comparison with the rotor axis is indicated by the angle V. This angle should have a certain size, for example 20° less than from the vertical as indicated.*

### 3. Selection of centrifuges and rotor assemblies where there is a risk of infection

3.1 The centrifuge outer casing, the control panel, the centrifuge chamber and the whole rotor assembly including the buckets should be easy to clean. The materials of construction should be able to resist the usual decontaminants, as well as alkaline chlorine-containing products [1, 12, 13].

3.2 To avoid formation of aerosols and leakage under centrifugation, the specimens in sealed tubes should be placed into sealed buckets or sealed rotor assemblies, i.e. the specimens are completely enclosed.

3.3 The temperature of a centrifuge will rise during centrifugation, due to air resistance, if no cooling is applied. The rise is generally less if an angle rotor or a wind shielded rotor assembly is used. Centrifuges which are cooled by inflow of air followed by exhaustion of this air into the laboratory, involve a risk of release of airborne infectious material when used for clinical samples. In such cases sealed buckets or sealed rotor assemblies should be used.

### 4. Electrical safety

Compliance with IEC [2] and local regulations will usually ensure a sufficient degree of electrical safety. However, the control panel should be so constructed that electrical accidents caused by ingress of moisture are prevented if the switches become splashed with materials handled in the laboratory. In laboratories where some of the centrifuges are not splash-proof, instruments which are provisioned should be especially marked. During laboratory procedures in which the risk of splashing is great, for example cell washing, splash-proof centrifuges must be used. Centrifuges which comply to the IEC 1010 part 1, general safety document [2] for laboratory instruments are splash-proof.

### 5. Centrifuges for flammable liquids

Centrifuges used for flammable liquids must be constructed to avoid fires and explosions. One approach is to avoid the use of electrical power altogether by using compressed air to run the rotor assembly, however, an out-of-balance switch may not be available. Another approach is to use spark-proof electrical components. A further possibility is to have a closed chamber which may be cooled and/or flushed by nitrogen.

### 6. Noise

6.1 Centrifuges usually add considerably to the noise level in the laboratory. A new centrifuge should be selected to minimize this problem.

6.2 The noise characteristics of an apparatus should be stated as the 'sound-power' level. The sound-power level is exclusively linked to the acoustic characteristics of the product, and not, as is the case for noise level (i.e. sound-pressure level), dependent on the measurement distance, position, mounting conditions, etc. The manufacturer may give information about the noise emitted by the centrifuge, expressed as a number called the dB(0)-value, which is a measure of the noise produced by the centrifuge in excess of an ideal noise level. dB(0)-values of 15–20 or less (at maximal rotational frequency) are generally acceptable. A brief report of how such data should be interpreted is available on request from Dr Uldall.

6.3 When the sound-power level of a centrifuge is not available, a rough comparison can be made using a simple sound level meter with a so-called A-weighting network. This A-weighting network operates in such a way that it balances the audible spectrum as it is perceived via the human ear; in this way the reading of the meter corresponds to the observer's estimate of the noise intensity through the ear [14].

### 7. Safety directions for the use of laboratory centrifuges

7.1 *Minimizing potential risk — choice of centrifuge for the actual work*

Practical experience has shown that the actual risk by working with laboratory centrifuges is very low; however, to reduce potential risk, the following precautions should be taken:

(*a*) To minimize the potential mechanical hazard, laboratory procedures should be chosen which requires the lowest possible kinetic energy. The smallest rotational frequency possible should be selected, and the shortest centrifugation time should be used. This applies for a certain separation with a given rotor assembly. Where two or more rotors are available one should select that with the smallest kinetic energy and which is sufficiently effective to do the job; this could be a rotor with a small mass, but utilizing a high rotational frequency.

(*b*) Preferably, large bench-top and floor-standing centrifuges should be placed in a separate room or in a separate section of the laboratory with reasonable distance between them and the other work stations in order to minimize the potential risk to people in the case of a disruption.

7.2 *Instruction manual*

A centrifuge must only be operated by people who have been fully instructed in its use, and the instruction manuals should be read carefully by the operator. The supplier's special instructions for operation and maintenance must be followed. The information which the manufacturer should provide is described by IEC [1, 2]. For every possible usage in laboratory, working instructions must be available. The contents of this instruction manual could be:

(*a*) Purpose.

(*b*) Reference to the local general centrifuge safety manual.

(*c*) Specifications of the centrifuge, for example brand, type, production year.

(*d*) Rotor, buckets, and accessories.

(*e*) Storage, special maintenance, and mounting of rotor, buckets and accessories.

(*f*) Counter balance technique.

(*g*) The settings of the centrifuge (rotational frequency, time, braking, temperature).

(*h*) Special conditions and precautions.

7.3 *General centrifuge safety manual*

General centrifuge safety instructions could be displayed as a poster near the centrifuge. Section 8 below shows the elements which should be included in a local general centrifuge safety manual. An assumption is made that infection risks always exist.

### 8. Elements of a general centrifuge safety manual

8.1 *Before starting the following precautions must be applied*

(*a*) For safe and efficient operation, be sure to carefully read the manual before using the centrifuge. Treat the manual carefully and keep it in a safe place close to the centrifuge.

(*b*) A safety clearance zone of 300 mm round the centrifuge must be observed [1]; requirements of fastening the centrifuge to the bench or floor must be considered.

(*c*) The centrifuge should be used in a room which is free from dust and dirt.

(*d*) Volatile flammable liquids give off vapour which can be explosive and must not be centrifuged without special safety precautions being taken. *Note:* The local health and safety authorities should be consulted. Permission to centrifuge flammable materials in closed containers may, for example be obtained if the centrifugation takes place in a separate room with remote control, or in a refrigerated centrifuge with suitable sealed buckets. However, centrifuges especially designed for the purpose are available, cf. section 5.

(*e*) Defective centrifuges must not be used.

(*f*) Any rotor or accessory of other type than described for the centrifuge should not be used except in case it is allowed by manufacturer.

(*g*) The rotor should always be checked to see if it is corroded. If any corrosion is found, the rotor should not be used unless the manufacturer permits it.

(*h*) When fitting or changing a centrifuge rotor great care must be taken to ensure that it is properly located on the rotor shaft and is properly tightened down according to the manufacturer's specification.

(*i*) Cracked, chipped, or otherwise damaged, glass and plastic tubes must not be used.

(*j*) Check that one, and only one, rubber insert is situated in the base of each tube holder.

(*k*) Check that closures of tubes are properly fitted to prevent loss of contents during centrifugation.

(*l*) Arrange the test-tubes evenly and symmetrically around, and in between the suspension projection of each bucket. Place any inserts in the buckets and check that the position in the buckets of any accessory liners is correct.

(*m*) Balance the buckets or multi-tube carriers together with sample tubes.

(*n*) Before closing sealed buckets and rotors, check that the seal is undamaged and placed correctly.

(*o*) Mount the buckets and carriers in pairs opposite to each other according to counter balance. Check that they are able to swing freely in their suspension mountings. Buckets must be mounted in all places in the rotor unless otherwise stated by the manufacturer.

(*p*) The rotor should never be rotated with any imbalance larger than the permissible one; in general the smaller imbalance the better, because the centrifuge vibrates less.

(*q*) Do not set a higher rotational frequency than specified maximum by the manufacturer for the load.

(*r*) Some centrifuges must have a slow acceleration.

8.2 *After starting it is essential to follow the three safety rules below*

(*a*) If the centrifuge is unstable and vibrates, is abnormally noisy, or shows any other sign of abnormality, stop it immediately and locate the cause.

(*b*) The centrifuge should never be moved while the rotor is rotating.

(*c*) No persons should stand close up to the centrifuge while the rotor is rotating unless it is needed for doing the job.

8.3 *After centrifugation observe the following points*

(*a*) Open sealed buckets in an exhaust protective cabinet.

(*b*) Buckets, re-usable tubes, rubber inserts and the centrifuge chamber must be cleaned immediately if liquids have been spilled or glass broken.

(*c*) Dispose of single-use items and broken glass carefully according to local safety guidelines.

(*d*) After cleaning and decontamination of the buckets/tube carriers their suspension mountings of the rotor must be greased with a high melting-point grease, for example SHELL Alvania Grease R3 which is lithium-based and has a solid degree of 3 (NLGI class 3) [18, 19].

(*e*) Clean regularly and with daily use, at least weekly. Buckets and tube carriers must be cleaned and decontaminated according to the manufacturer's instructions. When there is no manufacturer's recommended method of decontamination available, for example where the centrifuge is no longer made, the advice of the local microbiologists should be obtained [12, 13].* Water must never be allowed to enter the base of the chamber. Cleaning should take place immediately after centrifugation of corrosive materials. Knives, scrapers etc. which will damage surface must not be used. The centrifuge chamber must be wiped with a damp cloth. Because there is a risk of infection the manufacturer's manual and the local microbiologist should be consulted.

(*f*) Do preventive maintenance as described under section 9.

* Note. Proposals for decontamination of buckets and tube carriers includes soaking them for 1 h in a solution of Diversol BX or an equivalent chlorine product containing a substance concentration of active chlorine and bromine equivalent to approximate 0·3 mol/l (corresponding to '1%' calculated as chlorine). Aluminium cannot tolerate Diversol BX or other alkaline products. Equipment which is made of aluminium alloys can alternatively, with advantage, be placed to soak for 1 h in an aqueous solution of glutaraldehyde, substance concentration 0·2 mol/l (corresponding to '2%'). Following this they must be rinsed with water and if necessary washed with ordinary soap, or if necessary cleansed with an aqueous solution of hydrogen peroxide substance concentration 0·9 mol/l (corresponding to '1%'). Contamination may be 'disinfected' using an aqueous solution of ethanol (concentration of 13·5 mol/l, also called 'aethanolum dilutum' and corresponding to a mass concentration of 0·62 kg/l of ethanol in the solution). Drying is now undertaken. Drying ovens must only be used when manufacturer recommends them. The above materials for decontamination may also apply for use in the centrifuge chamber.

### 9. Preventive maintenance of centrifuges

The following points should be checked regularly, and at least annually:

9.1 *General subjects*

(*a*) Discussion with the operators regarding any running problems, accidents, breakdowns and repairs.

(*b*) Check that the local safety instructions and the manufacturer's operation instructions (in local language) are to be found with the centrifuge.

(*c*) Undertake all maintenance in accordance with the manufacturer's instructions. The points in section 9.2–9.3 should always be included.

(*d*) Enter all findings in the instrument's log book.

9.2 *Mechanical aspects of rotor assemblies, motor, casing, access cover*

(*a*) Make a visual check of all centrifuge rotors, buckets and tube carriers, for signs of corrosion, undue wear and tear, accident damage and cracks. Check any trunnion fastenings of the rotor and fastenings of the rotor to the motor shaft. Check the greasing.

(*b*) Check that the mounting of the centrifuge motor and chassis are intact (especially the rubber suspension vibration damper) and tightly secured. Check by gentle push and pull, that the rotor resistance is equal in all directions without there being too much play.

(*c*) Check, during running, that the rotor and buckets are in balance that there are no abnormal sounds, for example from the bearings.

(*d*) Check the access cover, hinges, springs and closing mechanism for wear and tear. Grease as necessary.

9.3 *Electrical parts*

(*a*) Check the electrical safety as described by local authorities (ref. [2, 20] will also provide most information). The electrical checks involve earth continuity, the cable insulation and plug. Check that a fuse with the rating specified by the manufacturer is fitted.

(*b*) Check the motor carbon brushes if possible, and change if necessary. Check the commutator. Remove any carbon dust.

(*c*) Clean the cooling fins.

(*d*) Check the safety interlock mechanism of the access cover. The centrifuge must not be able to start with the access cover open, neither must the access cover be able to be opened while the motor is running, or when the power is off (except for where $E_k < 1 kJ$).

(*e*) Check that the out-of-balance switch is working correctly (this may be described in the manufacturer's instructions).

(*f*) Check (whenever possible with a stroboscope) that the maximum allowed rotational frequency for each rotor cannot be exceeded.

(*g*) Check that the normal braking time does not exceed that stated by the manufacturer.

### Acknowledgements

Secretary Mrs Marianne Jensen is thanked for excellent assistance of the IFCC centrifuge safety working group in the preparation of the IFCC guidelines. The authors are indebted to mechanical technician Mr Jim Mikkelsen, who assisted in the series of provoked centrifuge disruptions, which provided important experience to the working group. Herlev Hospital is acknowledged for providing the facilities for the experiments, which also included invaluable assistance from the AV Department.

The manufacturers below and their representatives in Denmark are acknowledged for providing centrifuges and for funding the evaluation noise emitted from centrifuges: Beckman Instruments Inc. (US); DuPont Company, Instrument Products (USA); Fison Scientific Equipment (MSE) (UK); Heraeus Sepatech GmbH (Germany); Sigma Laborzentrifugen GmbH (Germany).

### References

1. IEC 1010 part 2, *Safety requirements – Electrical equipment for measurement, control and laboratory use. Part 2. Particular safety requirements for laboratory centrifuges. Committee draft (SC66E/WG3 (Secretariat) 41).* Geneva, Central Office of IEC 1990. 1–22 (the most recent version is a central office document No. 11, 15 April 1991).
2. IEC 1010 part 1, *Safety requirements – Electrical equipment for measurement, control and laboratory use.* IEC, Geneva (1990), 1–187.
3. ISO 9000, *Quality management and quality assurance standards – Guidelines for selection and use.* ISO, Geneva (1987), I–III & 1–6.
4. ISO 9001, *Quality systems – Model for quality assurance in design, development, production, installation and servicing capability.* ISO, Geneva (1987), I–III & 1–7.
5. ISO 9002, *Quality systems – Model for quality assurance in production and installation.* ISO, Geneva (1987), I–III & 1–6.
6. ISO 9003, *Quality systems – Model for quality assurance in production and installation.* ISO, Geneva (1987), I–III & 1–2.
7. ISO 9004, *Quality management and quality system elements – Guidelines.* ISO, Geneva (1987), I–III & 1–16.
8. Richwood, D. (Ed.), *Centrifugation: A Practical Approach.* IRL Press, London, Oxford, Washington, D.C. (1984), 1–352.
9. Uldall, A., Trier Damgaard, P., Magnussen, E., Drachmann, O. and Rigg, J. C., Guidelines (1988) for listing specifications of centrifuges. *Journal of Automatic Chemistry*, **11** (1989), 28–31.
10. British Standards Institution, Specification for safety requirements for laboratory centrifuges. BS 4402 (1982), 1–14.
11. Uldall, A., Trier Damgaard, P., Magnusen, E. and Jørgensen, F., Selection of laboratory centrifuges. Aspects of mechanical safety. *Annals of Clinical Biochemistry* (1987), 24 suppl. 2: 280 (a more detailed report on the experiences gained through experimental disruptions of centrifuges is available on request; so is a video).
12. Department of Health and Social Security, Code of practice for the prevention of infection in clinical laboratories and post-mortem rooms. Her Majesty's Stationery Office, London (1978), 23–25.

13. NCCLS M24-T, *Tentative guideline. Protection of laboratory workers from infectious diseases transmitted by blood, body fluids and tissue.* NCCLS Doc. (1989), 9, No. 1: 12–15, 26.
14. IEC 651, *Sound level meters.* IEC, Geneva (1979), 1–53.
15. ISO 3741, *Acoustics – Determination of sound power levels of noise sources – Precision methods for broad-band sources in reverberation rooms.* ISO, Geneva (1988), I–IV, 1–15.
16. ISO 3742, *Acoustics – Determination of sound power levels of noise sources – Precision methods of sound power levels of noise sound – Precision methods for discrete-frequency and narrow band sources in reverberation rooms.* ISO, Geneva (1988), I–IV, 1–8.
17. ISO 1969, *Acoustics – Assessment of noise with respect to community response.* ISO, Geneva (1969).
18. DIN 51825 Schmierstoffe, *Schmierfette. Schmierfette K im Gebrauchs-Temperaturbereich.* Teil 1. Beuth Verlag GmbH, Berlin (1981), 1–4 (revised edition: Entwürf, ibid, 1988: 1–8).
19. ISO 2137, *Petroleum products – Lubricating grease and petrolatum – Determination of case penetration.* ISO, Geneva (1985), 1–15.
20. IEC 601-1, *Medical electrical equipment. Part 1. General requirements for safety.* Bureau Central de la Commision Electrotechnique International/ISO, Geneva (1988), 1–351.

### Appendix A. Nomenclature of laboratory centrifuges

The parts of centrifuges should be named and described according to the list below. Some parts are also illustrated in figure 2 and figure 3.

*Access cover:* the lid of centrifuge which gives access to rotating parts.

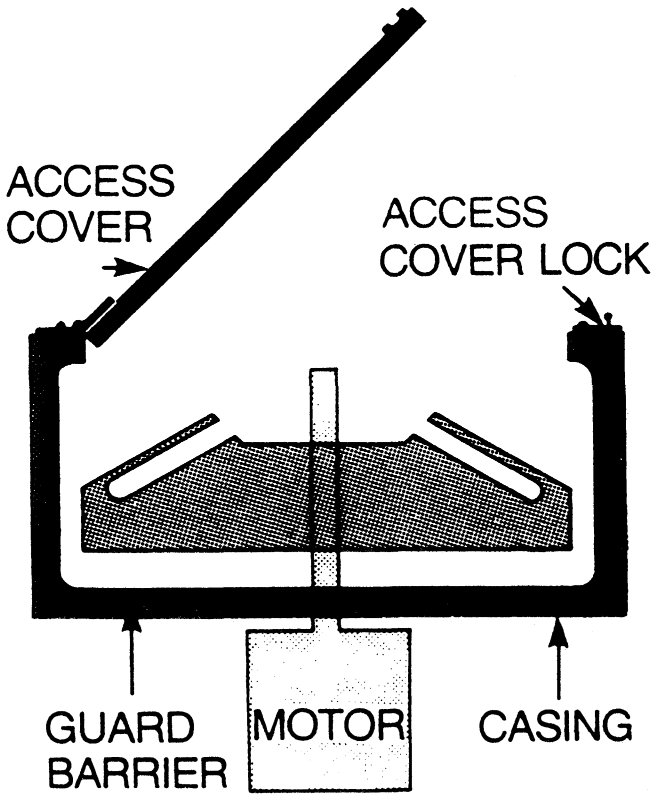

*Figure 2. Centrifuge with angle rotor.*

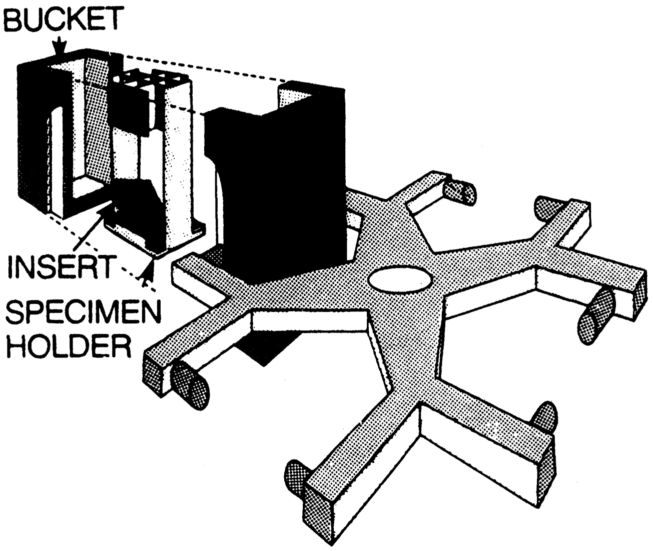

*Figure 3. Swing-out rotor and attachments.*

*Access cover lock:* safety lock or snap lock. In centrifuges of maximal kinetic energy usually less than 1 kJ, the snap lock switches off the power when it is opened.

*Adaptor:* a fitment allowing specimen holders of different sizes to be placed in the rotor assembly.

*Angle rotor:* a centrifuge rotor into which tubes or tube holders can be placed at an angle that is maintained during rotation.

*Bioseal:* a device or mechanism additional to, or integral with, a rotor or bucket and a closure assembly designed to prevent the escape of contents, for example microbiological material, during centrifugation.

*Brake:* mechanical or electrical device to reduce rotational frequency.

*Bucket:* rotor accessory designed to support one or more containers.

*Casing:* the cabinet of the centrifuge, including top access cover and case. Usually the casing includes a guard barrier or may surround a guard barrier, and may then be named protective casing.

*Centrifuge:* a motor-driven machine used in laboratories to separate components, or to alter the local distribution of components of a system by means of centrifugal acceleration in rapidly rotating vessels.

*Chamber:* the space enclosed by the casing of a centrifuge in which the rotor assembly rotates.

*Guard barrier:* a mechanically reinforced part of the casing or a separate strong shield surrounding the centrifuge chamber.

*Insert* (to specimen-holder assembly): special adaptors for different types of tubes, thus a certain specimen-holder assembly can be used for different tubes.

*Indicator* (on equipment): a device indicating the value of an operating variable for a piece of equipment, for instance rotational frequency of a centrifuge.

*Out-of-balance switch:* a switch to cut out the motor of a centrifuge that is out of balance.

*Rotational frequency selector:* an operating device for adjustment of rotational frequency.

*Rotor* (head, centre piece): the part of the centrifuge that is mounted directly to the rotating axis and rotates. Special types are the angle rotor (see figure 2) and the swing-out rotor (see figure 3). For the latter type, the buckets are not part of the rotor.

*Rotor assembly:* the centrifuge rotor with any buckets, tube holders, specimen holders, adaptors, inserts and lid for sealed rotors or buckets.

*Safety interlock:* a device, usually electrically operated, preventing opening of a centrifuge during operation. Safety interlocks are used mainly in centrifuges of maximum kinetic energy of the rotation assembly greater than 1 kJ.

*Sealed rotor assembly (or bucket):* special rotor assembly, or bucket, incorporating a bioseal of some centrifuges to reduce the hazard of aerosol production.

*Specimen-holder:* container or support which is placed inside a bucket to hold a specimen, for example a centrifuge tube.

*Specimen-holder assembly:* the complete assembly in which the specimens are housed.

*Swing-out rotor:* a centrifuge rotor in which the specimen holders increase their angle in relation to the axis of spin during rotation.

### Appendix B. Quantities and units commonly used in centrifuging

A quantity is a measurable physical (or chemical) property of a system. It can be expressed as a product of a numerical value and a unit: quantity = numerical value × unit. Table 2 shows the commonly used quantities in centrifugation. The terms below provide definitions for selected quantities:

*Acceleration.* Definition: rate of change in velocity.

*Acceleration due to gravity.* Definition: acceleration at a free fall in vacuum.

*Standard acceleration due to gravity.* Definition: acceleration due to gravity for 45° latitude ($g_n$ = 9·806 65 m s$^{-2}$).

*Centrifugal acceleration.* Definition: acceleration of a component in a centrifuge as a result of the rotational movement.

*Table 2. Quantities commonly used in centrifugation.*

| Name | Symbol | Unit |
|---|---|---|
| Radius | $r$ | m |
| Mass | $m$ | kg |
| Time | $t$ | s |
| Rotational frequency | $f_{rot}$ | Hz = s$^{-1}$ |
| Acceleration | $a$ | m s$^{-2}$ |
| Acceleration due to gravity | $g$ | m s$^{-2}$ |
| Standard acceleration due to gravity | $g_n$ | m s$^{-2}$ |
| Centrifugal acceleration | $a_{rot}$ | m s$^{-2}$ |
| Centrifugal force | $F_{rot}$ | N = kg m s$^{-2}$ |
| Kinetic energy | $E_k$ | J = kg m$^2$ s$^{-2}$ |

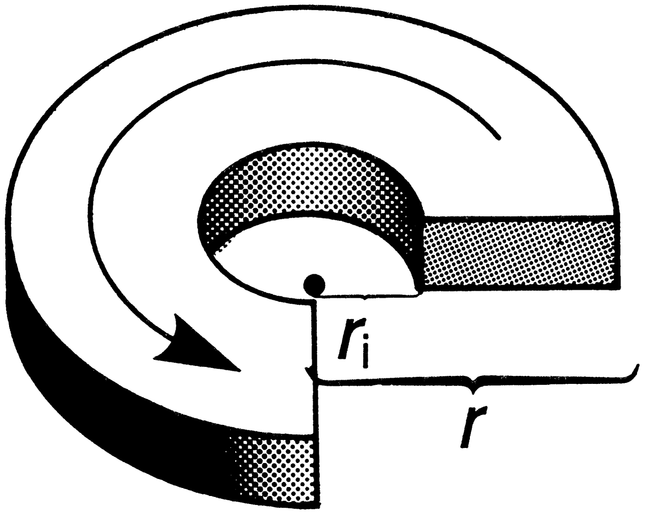

*Figure 4. Rotating ring with inner ($r_i$) and outer (r) radii.*

*Relative centrifugal acceleration,* cf. Appendix C.

*Rotational frequency.* Definition: number of rotations (or revolutions) divided by time. Synonyms are rate of rotation, rate of revolution, centrifugation speed. The preferred unit of rotational frequency is Hertz (Hz). The traditional unit: revolutions per minute (r.p.m., rev./min, r/min) is not recommended.

### Appendix C. Calculation of the centrifugal acceleration and kinetic energy of the rotor assembly

1.1 The *centrifugal acceleration* may be calculated from effective radius and rotational frequency: $a_{rot} = 4\pi^2 r f_{rot}^2$

Centrifugal acceleration is commonly expressed in terms of standard acceleration:

$1 \text{ m s}^{-2} = (1/9{\cdot}806\ 65)\ g_n$

$a_{rot} = (4\pi^2/9{\cdot}806\ 65)\ (r/\text{m})\ (f_{rot}/\text{Hz})^2\ g_n$

or *relative centrifugal acceleration,* $a_{rot}/g_n$

Table 3 shows the values of relative centrifugal acceleration at various rotational frequencies and is an aid for easy calculations.

1.2 *Example.*
Radius at which the component is spinning: $r$ = 170 mm
Rotational frequency: $f_{rot}$ = 50 Hz (= 50 s$^{-1}$ = 3000 min$^{-1}$)
$a_{rot} = (4\pi^2/9{\cdot}807)\ (170 \text{ mm/m})\ (50 \text{ Hz/Hz})^2\ g_n$
$a_{rot} = (4{\cdot}0257)\ (170 \times 0{\cdot}001)\ (50)^2\ g_n = 1711\ g_n$.

1.3 The centrifuge manufacturer usually calculates the acceleration of a rotor assembly for the bottom of the buckets. The user, however, knows the exact position of the meniscus of a liquid specimen and may calculate the centrifugal acceleration for top, bottom and mid position of the specimen.

*Table 3. Centrifugal acceleration for varying rotational frequencies at a fixed radius* r = *0·100 m. Accelerations for other radii may be calculated proportionally.*

| Rotational frequency | | Relative centrifugal acceleration |
|---|---|---|
| $f_{rot}$/Hz | $f_{rot}$/min$^{-1}$ | $10^{-3}\ a_{rot}/g_n$ |
| 5 | 300 | 0·010 |
| 10 | 600 | 0·040 |
| 15 | 900 | 0·091 |
| 20 | 1 200 | 0·161 |
| 25 | 1 500 | 0·252 |
| 30 | 1 800 | 0·362 |
| 35 | 2 100 | 0·493 |
| 40 | 2 400 | 0·644 |
| 45 | 2 700 | 0·815 |
| 50 | 3 000 | 1·01 |
| 60 | 3 600 | 1·45 |
| 70 | 4 200 | 1·97 |
| 80 | 4 900 | 2·58 |
| 90 | 5 400 | 3·26 |
| 100 | 6 000 | 4·03 |
| 120 | 7 200 | 5·80 |
| 150 | 9 000 | 9·06 |
| 200 | 12 000 | 16·1 |
| 250 | 15 000 | 25·2 |
| 300 | 18 000 | 36·2 |
| 350 | 21 000 | 49·3 |
| 400 | 24 000 | 64·4 |
| 450 | 27 000 | 81·5 |
| 500 | 30 000 | 101 |
| 600 | 36 000 | 145 |
| 700 | 42 000 | 197 |
| 800 | 49 000 | 258 |
| 900 | 54 000 | 326 |
| 1000 | 60 000 | 403 |

1.4 Separation involves sedimentation of material over a certain distance. Apart from centrifugal acceleration, the sedimentation velocity of particles is influenced by particle size, density of the particle and of the liquid phase, viscosity and temperature. Under centrifugation in an angle rotor a particle travels towards the tube wall and aggregates there with other particles. The aggregates move along the tube wall to the bottom; thus the travel is much faster than the migration of single particle due to increased mass. All such effects are much utilized in connection with ultracentrifuges which are not within the main scope of this publication. A handbook on the theory and practice of ultracentrifuges is available elsewhere (ref. [8] in the present main document).

2.1 The *kinetic energy* of a rotating body may be calculated by summing contributions from all partial masses $m_p$ of the body at distances $r_p$ from the axis of rotation:

$E_k = 2\pi^2 f_{rot}^2\, \Sigma(m_p\, r_p{}^2)$.

$\Sigma(m_p\, e_p{}^2)$ is called the moment of inertia of the body.

2.2 For a rotating uniform disc: $E_k = \pi^2\, m\, r^2 f_{rot}^2$.

2.3 For a rotating uniform ring, figure 4, with outer radius $r$ and inner radius $r_i$:

